# Impact of aluminum phosphide on development of the forensically important fly, *Chrysomya albiceps* (Diptera: Calliphoridae)

**DOI:** 10.1590/S1984-29612024006

**Published:** 2024-02-05

**Authors:** Mahran Tony, Abdullah Zahra, Nora Zeidan Abdellah, Abdelbaset Mohamed Ahmed Abdelreheem, Mohammad Reda Kamel Abdel-Samad

**Affiliations:** 1 Department of Zoology, Faculty of Science, Al-Azhar University, Assiut, Egypt; 2 Plant Protection Department, Faculty of Agriculture, Al-Azhar University, Cairo, Egypt; 3 Forensic Medicine and Clinical Toxicology, Faculty of Medicine, Assiut University, Assiut, Egypt; 4 Zoology and Entomology Department, Faculty of Science, Al-Azhar University, Cairo, Egypt

**Keywords:** Chrysomya albiceps, insecticides, forensic entomology, maggot, post-mortem interval, Chrysomya albiceps, inseticidas, entomologia forense, larva, intervalo pós-morte

## Abstract

*Chrysomya albiceps* (Calliphoridae) is among the earliest successional fauna on human and animal cadavers. Some immature Calliphoridae can be useful for determination of post-mortem interval. Toxins, particularly pesticides, can affect the rate of insect growth. Aluminum phosphide (AlP) is an affordable insecticide that has not been adequately entomotoxicologically evaluated. So, the impact of AlP on the developmental rate of different stages of *C. albiceps* was investigated. Larvae of *C. albiceps* were reared on the rabbit carcasses containing AlP as a treated group, and distilled water as a control group. The substances were administered by a gastric tube. The duration needed for development of *C. albiceps* stages was documented. Body length, width and weight of larvae were measured after 24, 48, 72 and 96 h from egg hatching. The duration of development increased significantly in the treated group compared to the control group. Larvae body measurements were significantly smaller in the treated group than in the control group. Therefore, it was demonstrated that AlP significantly influences the size of *C. albiceps* larvae and extends their development. During forensic application, interpretation of *C. albiceps* data should be used with caution when aluminum phosphide may be the cause of death.

## Resumo

*Chrysomya albiceps* (Calliphoridae) está entre as primeiras espécies a colonizar cadáveres humanos e animais. Alguns Calliphoridae imaturos podem ser úteis para a determinação do intervalo “post-mortem”. Toxinas, particularmente pesticidas, podem afetar a taxa de crescimento de insetos. O fosfeto de alumínio (AlP) é um inseticida acessível que não foi adequadamente avaliado entomotoxicologicamente. Assim, o impacto do AlP na taxa de desenvolvimento de diferentes estágios de *C. albiceps* foi investigado. Larvas de *C. albiceps* foram criadas em carcaças de coelho, contendo AlP como grupo tratado e água destilada como grupo controle. As substâncias foram administradas por sonda gástrica. A duração necessária para o desenvolvimento dos estágios de *C. albiceps* foi documentada. O comprimento, largura e peso do corpo das larvas foram medidos após 24, 48, 72 e 96 horas da eclosão dos ovos. A duração do desenvolvimento foi significativamente aumentada no grupo tratado, em comparação com o grupo controle. As medidas corporais das larvas foram significativamente menores no grupo tratado do que no grupo controle. Portanto, foi demonstrado que o AlP influencia significativamente o tamanho das larvas de *C. albiceps* e prolonga seu desenvolvimento. Durante a aplicação forense, a interpretação dos dados de *C. albiceps* deve ser usada com cautela, quando o fosfeto de alumínio puder ser a causa da morte.

## Introduction

Suicide causes the deaths of over 700,000 persons each year, and it is the fourth greatest reason for death among persons aged 15 to 29. Seventy-seven percent of suicides worldwide occur in low- and middle-income nations and pesticide ingestion is one of the most popular suicide ways worldwide (WHO, 2021).

Some pesticides such as aluminum phosphide (AlP) are used for protecting crops during storage and shipping (Bumbrah et al., 2012). Accidental and suicidal human toxicity with AlP in most cases ends with death. Among the most critical aspects of death investigation is determining the cause of death (Chophi et al., 2019).

In cases of death by poisoning and when a body reaches advanced putrefaction, usually there are no available tissues for toxicological analysis, but insects that have fed on the body might be a potential proxy material for analysis in these cases (Nolte et al., 1992). Where, the insects can colonize the dead bodies in succession waves as putrefaction begins (Byrd & Tomberlin, 2019). Furthermore, the presence of pharmaceutical preparations and toxic substances in decomposing cadavers might affect the rates of development of insect larvae that consumed the deceased bodies. This might change the time required for larval development and potentially introduce errors when estimating the time since death, i. e. the post-mortem interval (Campobasso et al., 2019).

The blow fly *C. albiceps* is a cosmopolitan species of the Calliphoridae family. Its larvae feed on decaying organic matter (carcasses, corpses, waste, and fecal matter), appearing early on corpses and reproducing quickly (Whitworth, 2006; Vasconcelos et al., 2016; Badenhorst & Villet, 2018). Some species of Calliphoridae may play an important role in forensic medicine being useful for estimating the time elapsed since death (the post-mortem interval) helping legal investigations (Hassan et al., 2013; Byrd & Tomberlin, 2019).

Further entomotoxicological studies are needed for accumulating data on the effects of various medications and toxins on a variety of forensically important species. Some studies were conducted on *C. albiceps* but the effect of the toxins on this species is still poorly known. The present study aimed to investigate the effect of aluminum phosphide on the development of immature stages of *C. albiceps*.

## Materials and Methods

### Origin and laboratory maintenance of flies

According to Hall (1995), rotten chicken viscera were used as bait for collecting adult *Chrysomya albiceps* flies. Recognition and taxonomic classification of flies were conducted by applying taxonomic keys to identify blow fly genera and species (Smith, 1986).

*Chrysomya albiceps* flies were reared for several generations under controlled laboratory conditions according to previous studies (Hassan et al., 2014; Tony et al., 2022), with minor modifications. In brief, The flies were kept in wooden cages (40 x 40 x 40 cm) supplied with required diet (Milk powder, 10% sugar solution, and liver slices) under temperature 25 ± 2 °C, relative humidity 60 ± 10%, and 12: 12 h light-dark regime.

### Animal model

This study was performed on six adult male New Zealand rabbits, weighing about 1.5 kg. The rabbits were accommodated in appropriate cast steel cages for 15 days for acclimatization at 25 ± 2 ºC, 12:12 h light-dark regime and relative humidity of 60 ± 10%. All animals stayed under similar conditions of temperature, light, noise, and ventilation, and received identical diets (vegetables, feed, and water) throughout the experimental period. The laboratory animal care and use guidelines were followed for animal housing, care, and procedures (National Research Council, 2011), after approval of the ethical committee.

### Experimental design

After acclimation, the rabbits were randomly distributed in two equal groups (control and treated). In the control group the rabbits received sterile water, while those in the treated group received AlP (32.8 mg/kg of body weight), both by intragastric route using a gastric tube (Tony et al., 2023). The rabbits were euthanized according to the American Veterinary Medical Association (AVMA) guidelines for the euthanasia of animals (Underwood & Anthony, 2013.

After euthanizing the rabbits, each carcass was dissected to facilitate the observation, put into a plastic box (25×10×15 cm), and placed in a cage containing 20 flies (13 females and 7 males) for 24 h. Eggs hatching was checked, and the time required for eggs hatching, larval development periods, pupation, pre-oviposition (from emergence to oviposition), and the whole period from egg to adult were recorded. The number of eggs and all stages were examined and counted using binocular dissecting microscope (Elshehaby et al., 2019). In addition, the morphological parameters (length and width) and weight of *C. albiceps* maggots were measured on random samples from each group after 24, 48, 72, and 96 h from egg hatching (Elshehaby et al., 2019; Bhardwaj et al., 2020).

### Statistical analysis

The significance of results from control and treatment groups was determined by using a two-tailed independent Student’s t-test using GraphPad Prism version 8.0.0 for Windows, GraphPad Software, San Diego, California, USA. The statistical significance level was chosen at p ˂ 0.05. Data were expressed as the mean ± standard error of mean.

## Results

**Figure 1:**
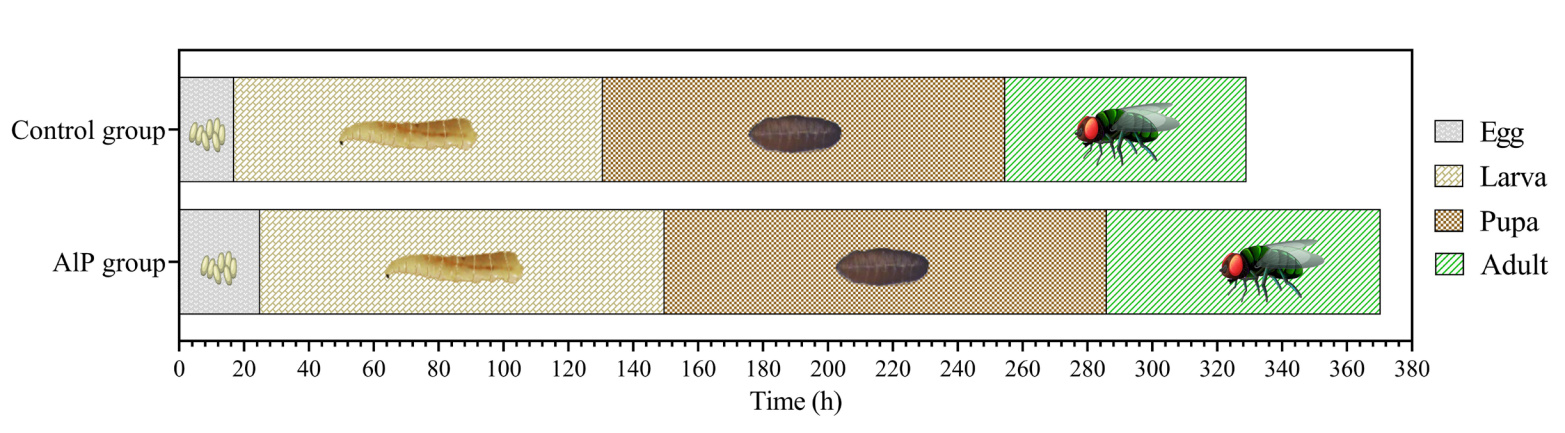
Influence of aluminum phosphide (AlP) on timeline development of *Chrysomya albiceps* in rabbit carcasses.

The development timelines of *Chrysomya albiceps* in the control and treated (AlP) groups were compared (Figure 1). Overall, AlP increased the time of *C. albiceps* development in treated group significantly by 12.46% compared to the control group. The average period for egg hatching in the control group was 17 h, reaching 25 h in the AlP group. The average time for larval development was 113.66 and 124.67 h for the control and AlP groups, respectively. The adults emerged after 124.00 and 136.33 h in control and AlP groups, respectively. The time for emerging adults to lay eggs in control group was 74.56 h, while it was 84.33 h in AlP group.

Moreover, the numbers of eggs, larvae, pupae and adults in this generation for the control and AlP groups were counted and shown in Table 1. All stages of *C. albiceps* were affected by the presence of AlP in treated rabbit carcasses. Significant decrease was detected in the numbers of eggs, larvae, pupae and adults of the treated group compared to the control group.

**Table 1:** Mean absolute frequency of development stages of *Chrysomya albiceps* reared in carcasses of rabbits previously treated with aluminum phosphide (AIP).

**Group**	**Egg No. (Mean±SEM)**	**Larval No. (Mean±SEM)**			**Pupae No. (Mean±SEM)**	**Adults No. (Mean±SEM)**
		1^st^ instar	2^nd^ instar	3^rd^ instar		
**Control**	287.33±2.40	269.33±3.48	242.00±2.65	233.00±2.08	212.00±1.53	194.33±1.76
**AlP**	222.00±3.21	206.67±1.86	189.67±1.33	167.00±2.65	156.67±2.91	137.00±4.73

SEM: standard error of mean.

**Figure 2:**
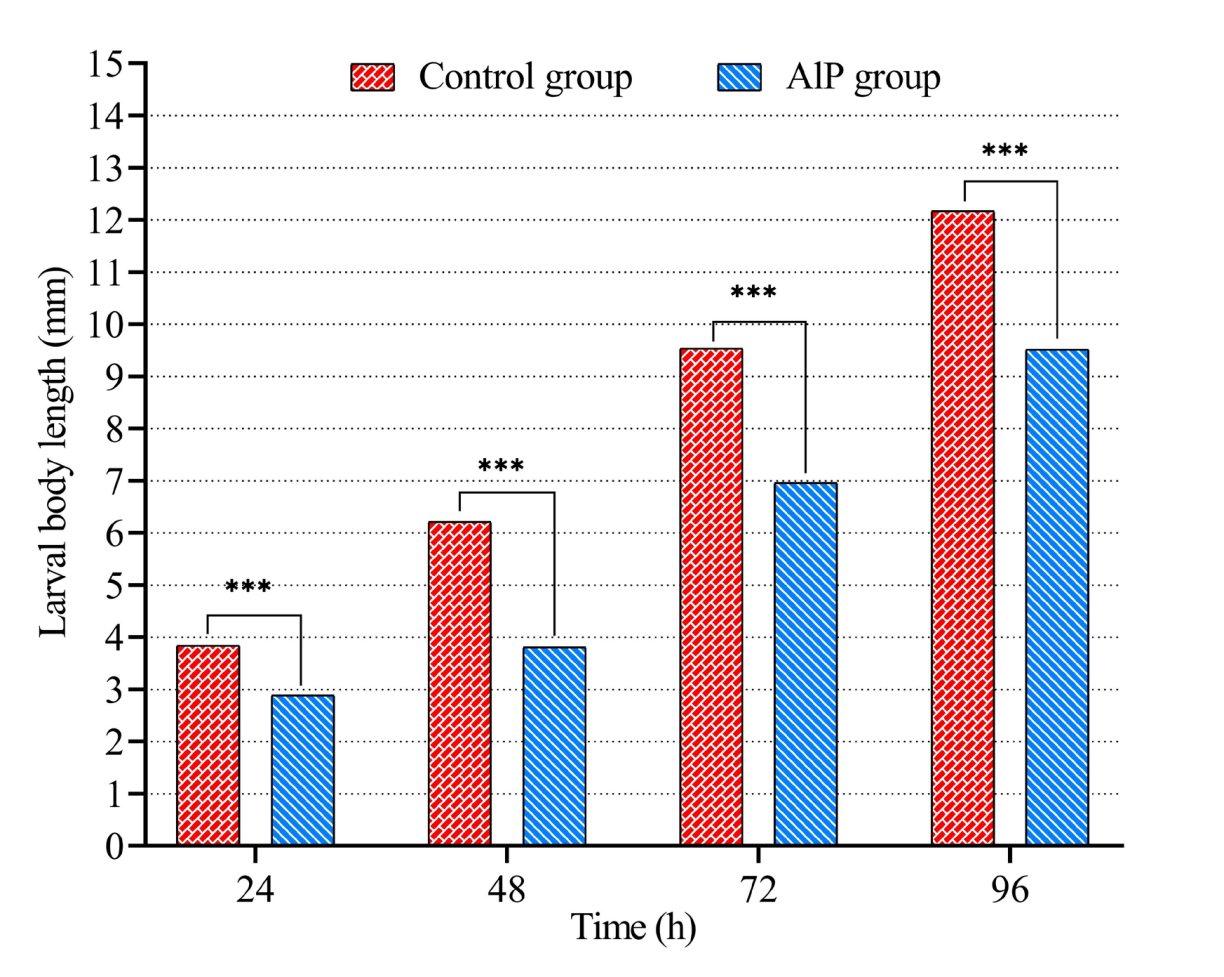
Length of *Chrysomya albiceps* larvae developed in carcasses of rabbits previously treated with aluminum phosphide (AlP). *** p < 0.001.

**Figure 3:**
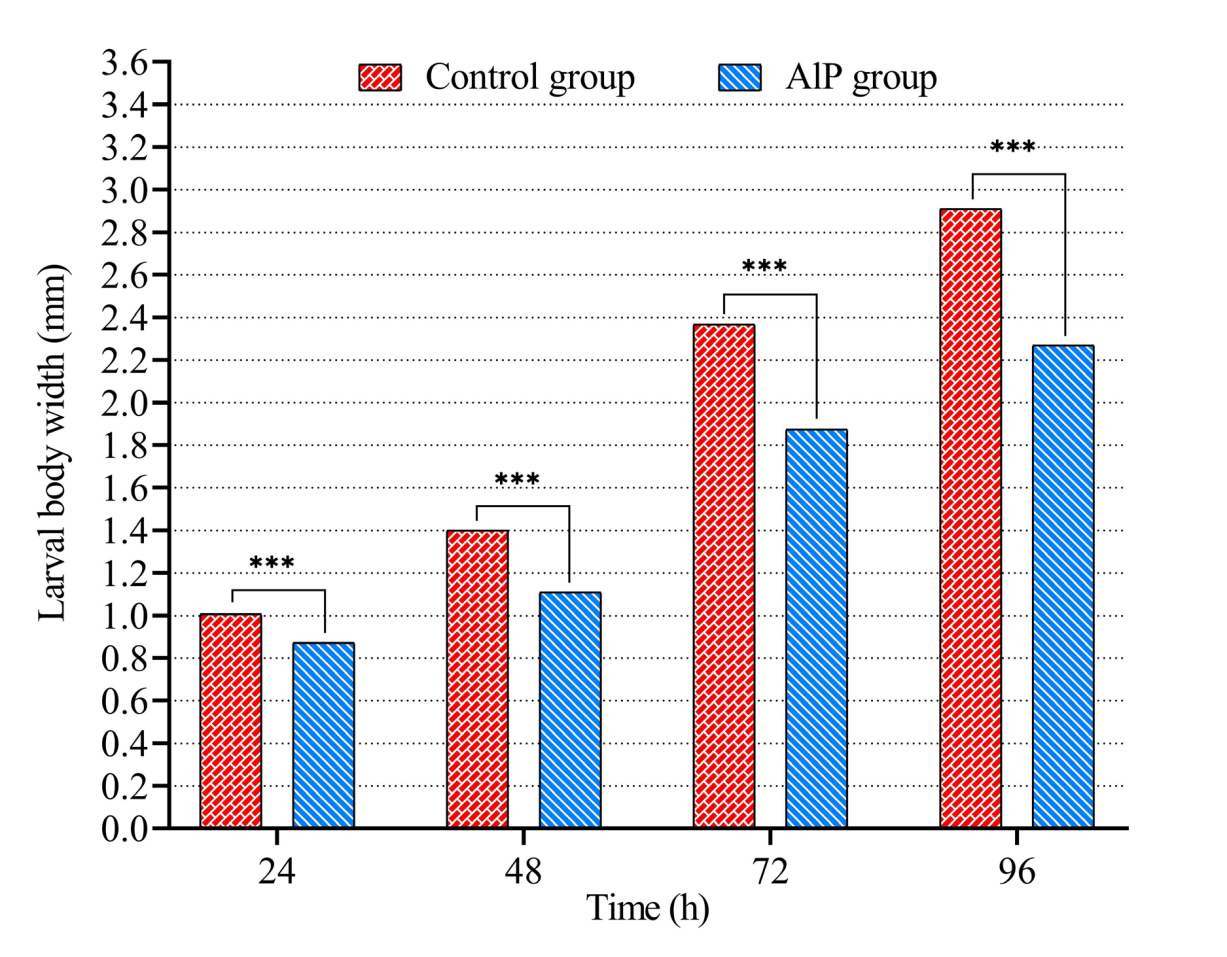
Width of *Chrysomya albiceps* larvae developed in carcasses of rabbits previously treated with aluminum phosphide (AlP). *** p < 0.001.

Also, the length, width, and weight of *C. albiceps* larvae were influenced by the presence of AlP in carcasses. On one hand, the mean larval body length increased gradually with larval growth until the end of feeding phase of third-stage larvae. A significant decrease was detected in length of larvae in AlP group compared to the control group (Table 2 and Figure 2). The larval body length in AlP group was 2.89 ± 0.02 mm at 24 h and 9.52 ± 0.07 mm at 96 h after hatching, while in the control group it was 3.84 ± 0.03 mm at 24 h and reached a final length of 12.17 ± 0.04 mm after 96 h. The width was also significantly smaller in larvae from the AlP group than in the control group (Table 2 and Figure 3). After 96 h, the maximum width of larvae in AlP group reached 2.27 ± 0.05 mm, while it achieved 2.91 ± 0.03 mm in control group.

**Table 2:** Length, Width and Weight of *Chrysomya albiceps* maggots reared in carcasses of rabbits previously treated with aluminum phosphide (AIP).

Time (h)	**Length (mm)** (Mean±SEM)		**Width (mm)** (Mean±SEM)		**Weight (mg)** (Mean±SEM)	
	Control	AlP	Control	AlP	Control	AlP
24	3.84±0.03	2.89±0.02	1.01±0.02	0.87±0.01	4.61±0.12	2.41±0.06
48	6.22±0.06	3.81±0.03	1.40±0.04	1.11±0.03	6.26±0.06	4.09±0.07
72	9.54±0.03	6.96±0.05	2.37±0.04	1.87±0.04	23.40±0.15	14.95±0.10
96	12.17±0.04	9.52±0.07	2.91±0.03	2.27±0.05	60.96±0.67	48.99±0.30

SEM: standard error of mean.

**Figure 4:**
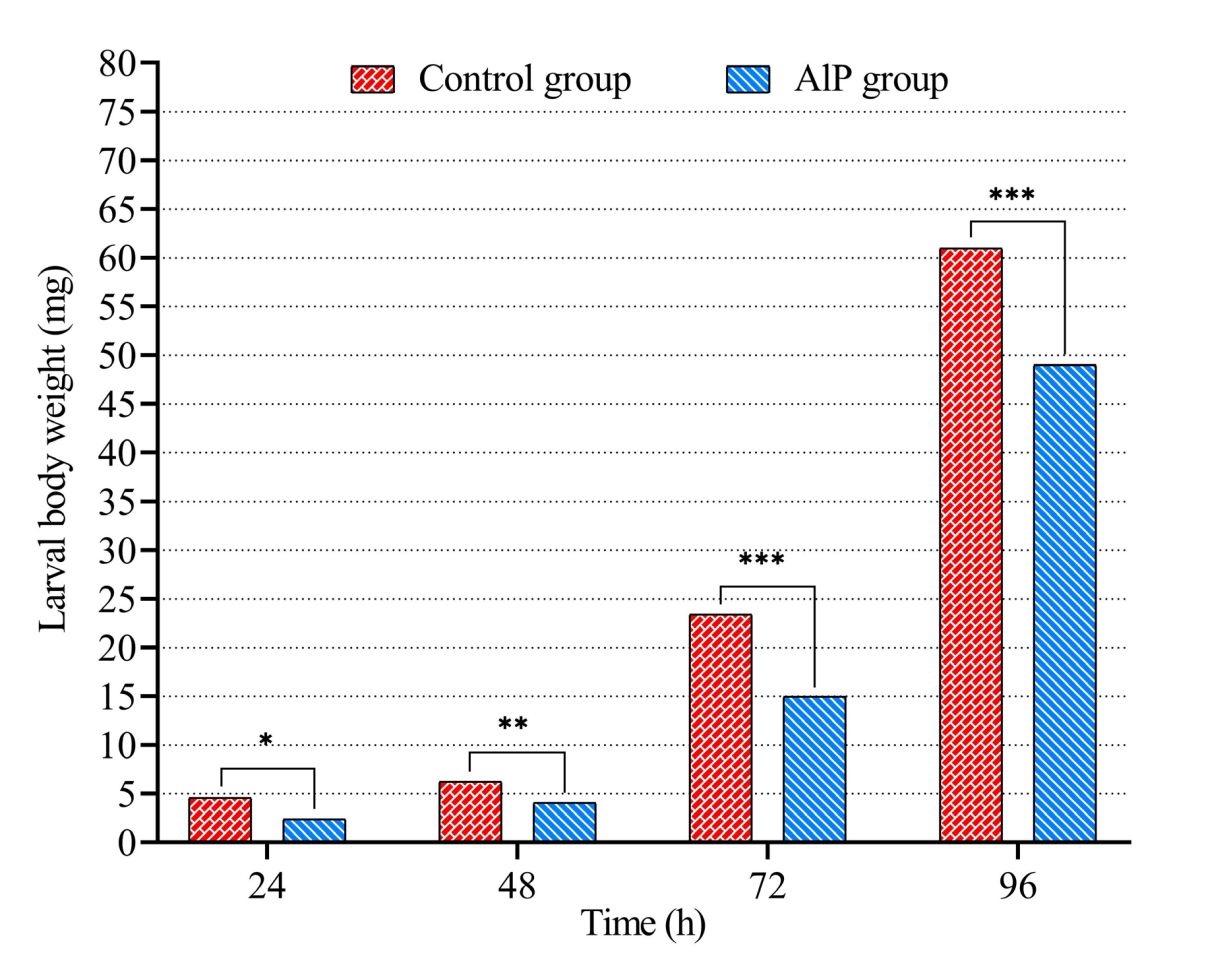
Weight of *Chrysomya albiceps* larvae developed in carcasses of rabbits previously treated with aluminum phosphide (AlP). * p < 0.05; ** p < 0.01; *** p < 0.001.

In addition, the larval body weights increased by the time with feeding. The weights of larvae that fed on carcasses of treated rabbits with AlP were affected and significantly decreased compared to larvae that fed on control carcasses (Table 2 and Figure 4). Such difference reached 19.63% after 96 h, when larval weight was 60.96 ± 0.67 and 48.99 ± 0.30 mg in the control and AlP groups, respectively.

## Discussion

Post-mortem interval (PMI) is the time elapsed from death until the discovery of dead bodies (Bhardwaj et al., 2020). At present, estimation of the PMI utilizing entomological data is broadly accepted, either by estimating the age of the oldest insects found on the corpse or by analyzing the composition of insect species found.

In addition, insects could act as valid toxicological samples when human biological samples are absent or invalid for analysis when in advanced decomposition. Several toxic substances present in corpses can be identified in insects, since the toxins are ingested during insect feeding on decomposing tissues (Amendt et al., 2010). Also, the concentrations of toxins in insects are related and comparable to the concentrations in tissues of dead bodies, thus providing helpful data to clarify the cause of death (Tracqui et al., 2004; Galil et al., 2021).

It is well settled that the presence of toxins in the decomposing tissues might change the rate of development of insects, resulting in an inaccurate estimation of the time of death (Amendt et al., 2011). So, the study of the effects of toxins and insecticides on different insect species can help clarify some issues in legal investigations. Therefore, the present study was conducted to investigate the impact of aluminum phosphide on the developmental rate of different stages (egg, larvae, pupa and adult) of *C. albiceps.*

The results showed a significant increase in *C. albiceps* development time of larvae fed on carcasses of rabbits treated with AlP. These results are supported by the work of Day & Wallman (2006) who revealed that the consumed diet is an important element affecting the growth and development of green-bottle flies, and agree with the study of El Hadi Mohamed et al. (2021), which demonstrated that diet shows a significant effect on the developing stages of *C. albiceps*. In agreement with the present results, AlP caused delay of growth of the larvae of *C. albiceps* (El-Ashram et al., 2022).

Previous studies have demonstrated different effects of toxins on the development rate of several fly species causing acceleration or retardation of their growth. Thus, codeine at different concentrations was reported to stimulate the growth of *Lucilia sericata* larvae reared in pig liver (Kharbouche et al., 2008), while morphine slowed their development rate (Bourel et al., 1999) and a longer development was observed in rabbits treated with tramadol (El-Samad et al., 2011). Also, heroin was reported to accelerate the development of *Boettchersica peregrina* (Goff et al., 1991).

Salimi et al. (2018) demonstrated possible underestimation of PMI based on the faulty elucidation of the development of *C. albiceps* larvae reared on carcasses containing morphine. The disparity among these studies can be delineated by some considerations such as food type, concentration of dietary ingredients, insect species and nature of used toxins.

Additionally, the survival rate of *C. albiceps* varied significantly between the control group and treated groups. Among all test groups, the control had the highest survival rate in all stages of *C. albiceps*.

The present study showed a significant variation in weight and morphometric parameters (length and width) of *C. albiceps* larvae reared in carcasses of animals treated with AlP. The average length, width and weight of larvae that fed in the treated group were significantly lower compared to the control. The present results are supported by the study of El-Ashram et al. (2022) who reported that *C. albiceps* flies that reared on AlP administered rabbits’ carcasses had a significantly lower gain in body length compared to control carcasses. Other toxins have different effects on the size of insects; ketamine was reported to have no significant effect on the larvae's length and weight (Zou et al., 2013).

## Conclusion

Aluminum phosphide in carcasses significantly increases the development period of *C. albiceps*, leading to a delay in growth and reduction of weight and morphometric parameters of larvae. Such effects may lead to errors in postmortem interval estimates based on larval development. Therefore, caution is advised in the application and interpretation of entomological data in situations where aluminum phosphide may be the cause of death.
